# Concurrent validity and test-retest reliability of the Virtual Peg Insertion Test to quantify upper limb function in patients with chronic stroke

**DOI:** 10.1186/s12984-016-0116-y

**Published:** 2016-01-22

**Authors:** Bernadette C. Tobler-Ammann, Eling D. de Bruin, Marie-Christine Fluet, Olivier Lambercy, Rob A. de Bie, Ruud H. Knols

**Affiliations:** Directorate of Research and Education, Physiotherapy & Occupational Therapy Research, University Hospital Zurich, Zurich, Switzerland; Department of Epidemiology, CAPHRI School for Primary Health and Primary Education, Maastricht University, Maastricht, The Netherlands; Institute of Human Movement Sciences and Sport, Department of Health Sciences and Technology, ETH Zurich, Wolfgang-Pauli-Str. 27, 8093 Zurich, Switzerland; Centre for Evidence Based Physiotherapy, Maastricht University, PO Box 616, 6200 MD Maastricht, The Netherlands; Rehabilitation Engineering Lab, Department of Health Sciences and Technology, ETH Zurich, Zurich, Switzerland; ReHaptix GmbH, ETH Zurich, Zurich, Switzerland

**Keywords:** Virtual Peg Insertion Test, Upper limb function, Stroke, Concurrent validity, Test-retest reliability

## Abstract

**Background:**

Measuring arm and hand function of the affected side is vital in stroke rehabilitation. Therefore, the Virtual Peg Insertion Test (VPIT), an assessment combining virtual reality and haptic feedback during a goal-oriented task derived from the Nine Hole Peg Test (NHPT), was developed. This study aimed to evaluate (1) the concurrent validity of key outcome measures of the VPIT, namely the execution time and the number of dropped pegs, with the NHPT and Box and Block Test (BBT), and (2) the test-retest-reliability of these parameters together with the VPIT’s additional kinetic and kinematic parameters in patients with chronic stroke.

The three tests were administered on 31 chronic patients with stroke in one session (concurrent validity), and the VPIT was retested in a second session 3–7 days later (test-retest reliability). Spearman rank correlation coefficients (ρ) were calculated for assessing concurrent validity, and intraclass correlation coefficients (ICCs) were used to determine relative reliability. Bland-Altman plots were drawn and the smallest detectable difference (SDD) was calculated to examine absolute reliability.

**Results:**

For the 31 included patients, 11 were able to perform the VPIT solely via use of their affected arm, whereas 20 patients also had to utilize support from their unaffected arm. For *n* = 31, the VPIT showed low correlations with the NHPT (*ρ* = 0.31 for *time* (T_ex_[s]); *ρ* = 0.21 for *number of dropped pegs* (N_dp_)) and BBT (*ρ* = −0.23 for *number of transported cubes* (N_tc_); *ρ* = −0.12 for *number of dropped cubes* (N_dc_)). The test-retest reliability for the parameters T_ex_[s], *mean grasping force* (F_g_go[N]), *number of zero-crossings* (N_zc[1/s_go/return) and *mean collision force* (F_cmean_[N]) were good to high, with ICCs ranging from 0.83 to 0.94. Fair reliability could be found for F_g_return (ICC = 0.75) and *trajectory error* (E_traj_go[cm]) (0.70). Poor reliability was measured for E_traj_return[cm] (0.67) and N_dp_ (0.58). The SDDs were: T_ex_ = 70.2 s, N_dp_ = 0.4 pegs; F_g_go/return = 3.5/1.2 Newton; N_zc[1/s]_go/return = 0.2/1.8 zero-crossings; E_traj_go/return = 0.5/0.8 cm; F_cmean_ = 0.7 Newton.

**Conclusions:**

The VPIT is a promising upper limb function assessment for patients with stroke requiring other components of upper limb motor performance than the NHPT and BBT. The high intra-subject variation indicated that it is a demanding test for this stroke sample, which necessitates a thorough introduction to this assessment. Once familiar, the VPIT provides more objective and comprehensive measurements of upper limb function than conventional, non-computerized hand assessments.

## Background

Upper limb function relies on the delicate interaction between hand and brain, defining our ability to perform activities of daily living (ADL) [1]. Brain injury caused by cerebrovascular accident may result in reduced cerebral hand representation over time [1]. The consequences for patients after a stroke are tremendous and ADL become challenging. However, building on brain plasticity and intensive training, hand representation in the brain can be increased again [2]. Novel upper limb training modalities include the use of virtual reality (VR), robotics and computer gaming, all of which can provide ecologically valid, intensive and task specific training [3]. It is essential to regularly measure motor function in order to document changes in upper limb performance during the course of rehabilitation. Such regular evaluation also informs adaptation of therapy settings, as needs arise. To date, there are several assessments measuring upper limb motor function, such as the Box and Block Test (BBT) [4, 5] and the Nine Hole Peg Test (NHPT) [6, 7] which measure gross and fine manual dexterity respectively. Additionally, the use of VR and robotic devices are not only an effective alternative to conventional therapy [8, 9], but can also be used for precise and objective concurrent assessment of attributes such as motor function, cognition and ADL [10–16].

A promising assessment tool for measuring upper limb function is the Virtual Peg Insertion Test (VPIT), a computer-assisted assessment. The task is that of the NHPT, but since there is no precision grip required, the movement has more similarities to the BBT [17]. The VPIT allows measurement of three-dimensional hand position and orientation as well as grasp force during the accomplishment of a goal-oriented task consisting of grasping, transporting and inserting nine virtual pegs into the nine holes of a virtual pegboard. This is achieved by positioning and controlling a grasping force applied to grasping a handle instrumented with force sensors mounted on a PHANTOM Omni haptic device (Geomagic, USA). The clinical practicability and measurement properties of the VPIT were tested in patients with Multiple Sclerosis (MS) [18] and pilot-tested in patients with Autosomal Recessive Spastic Ataxia of Charlevoix-Saguenay (ARSACS) [19]. Both patient groups were significantly less coordinated and slower than age-matched healthy subjects when evaluated using the VPIT. The preliminary results of both studies illustrate the feasibility of using the VPIT in both MS and ARSACS patients, and underline the potential of this test to evaluate upper limb motor function. To date, there is only one pilot study reporting initial evaluation of the VPIT outcome measures in a group of four chronic patients with stroke, showing significant differences in grasping force control and upper limb movement patterns compared to healthy subjects [17]. However, the relation between the analyzed performance parameters during the VPIT and impaired function needs to be further established. A way to achieve this would be to evaluate the validity and reliability of the VPIT parameters for this population. As the VPIT combines the characteristics of the conventional NHPT and the BBT, one option could be to validate their mutual parameters, namely the execution time (T_ex_[s]) and the number of dropped pegs (N_dp_). As the VPIT provides in total nine different outcome measures including kinematic and kinetic parameters quantifying movement coordination, smoothness, upper limb synergies and force control, we decided to evaluate all of these for their reliability when administered twice in a test and retest procedure. Therefore, this study aimed to evaluate (1) the concurrent validity of key outcome measures of the VPIT, namely the execution time and the number of dropped pegs, with the NHPT and BBT, and (2) the test-retest reliability of these parameters together with the VPIT’s additional kinetic and kinematic parameters, in patients with chronic stroke.

## Methods

### Apparatus

**Fig. 1:**
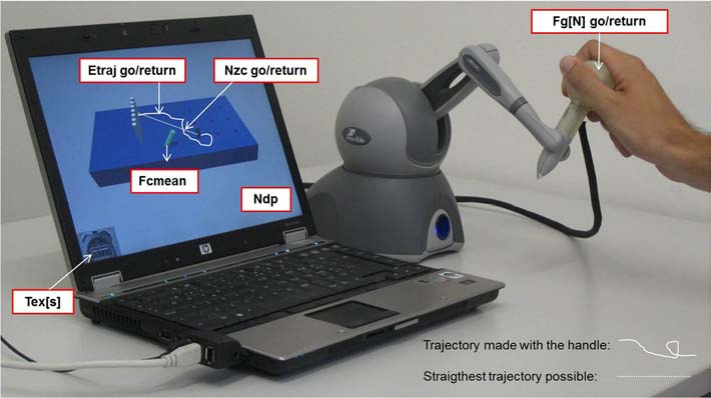
VPIT setup and upper limb function parameters. T_ex_[s]: execution time in seconds; N_dp_: number of dropped pegs during transport. Kinematic parameters: E_traj_[cm]go/return: trajectory error: Nzc[1/s]go/return: number of zero-crossings of the acceleration. Kinetic parameters: F_g_[N]go/return: mean grasping force; F_cmean_[N]: mean collision force

In the VPIT, pegs and holes are displayed in a virtual environment and can be felt through a haptic interface. Subjects are asked to move the handle of the haptic device to grasp, displace and release the pegs (Fig. 1). The aim is to grasp, transport and place all pegs in the holes as fast as possible. To evaluate upper limb function during the execution of the task, the VPIT software records and computes nine specific parameters. These are (Fig. 1):

The two primary outcome parameters evaluating the overall arm and hand functional ability of the patient are: (1) Tex[s]: time to execute the task from the approach to the first peg to the insertion of the last peg. (2) Ndp: number of times a peg is dropped during the transport. Secondary outcome parameters pertain to four kinematic and three kinetic parameters.

The four kinematic parameters are: (3–4) Etraj[cm]go/return: trajectory error measured as the distance between the actual trajectory and the ideal straightest trajectory projected on the horizontal plane. This parameter is used to evaluate movement accuracy and upper limb motor synergies during a movement requiring the simultaneous control of shoulder, elbow and hand [20–22]. (5–6) Nzc[1/s]go/return: the time-normalized number of zero-crossings (i.e., change of sign) of the acceleration during the gross movement from a peg to a hole, or from a hole to a peg. This parameter provides an estimate of the number of sub-movements a point-to-point movement is composed of, which is a commonly accepted measure of movement smoothness and upper limb coordination [19, 23, 24].

The three kinetic parameters are: (7–8) F_g_[N]go/return: average grasping force calculated during the transport of one peg and for the return trajectory. This parameter evaluates factors such as force control. (9) F_cmean_[N]: mean collision force exerted against the virtual pegboard.

The “go” refers to the trajectory during the outbound trip when a peg is transported to the hole, and the “return” stands for the way back from the hole to approach a new peg. While performing the VPIT, the patient additionally receives visual feedback regarding the force applied on the peg being held: the cursor is yellow when no peg is held, turns orange to indicate that it is properly aligned with a peg, green when a peg is currently held and red when excessive grasping force is applied to the handle but no peg is held. Parameters are computed offline and no feedback related to the user’s performance is displayed during or after the test. For a more detailed description of the apparatus, we refer to Fluet et al. (2011) [17].

### Conventional tests

The NHPT version produced by Smith & Nephew Rehabilitation, Inc.^1^ was used. It consists of a plastic board with a shallow round dish to contain the pegs on one end of the board and the nine holes in a 3x3 grid on the opposite end. Initially, the participant had to use their affected hand to grasp, one by one, the nine pegs from the dish, inserting each one into a hole until all pegs are placed. After this the participant then has to replace each of the pegs back into the dish. All of this is carried out as fast as possible [7]. The test was timed, with a stopwatch, from the moment the participant touched the first peg until the moment when all pegs were removed from the holes. As the VPIT stops when all pegs are placed into the holes, we measured an intermediate time when all pegs were placed into the holes of the NHPT board. We labelled this parameter “time point 1” (TP1). All dropped pegs were counted and noted on the case report form (CRF).

The BBT consists of 50 wooden blocks (cubes of 2.54 cm on each side) placed in a wooden box that has 2 equal-sized compartments that are separated by a central wooden partition of 15.2 cm height [5]. The participant was instructed to use their affected hand to move blocks, one by one, from one compartment to the other, evaluating the maximum number which could be transferred within 1 min. The transported blocks were counted during the test duration with the help of a counter clicker. All dropped blocks were noted on the CRF.

### Participants

In total, 31 patients with stroke were consecutively recruited in this study. The occupational therapy (OT) outpatient practices that are registered as specialized in neurorehabilitation in the canton of Bern, Switzerland, were contacted to recruit patients with chronic stroke. The inclusion criteria were (1) a stroke diagnosis at least 6 months before study inclusion confirmed by a physician, (2) ≥ 18 years of age, (3) ability to communicate in German language, (4) capable of sitting in a (wheel-)chair with a backrest for up to 90 min, (5) able to lift and hold the arm in 90° of elbow flexion and 45° of shoulder abduction and (6) able to grasp a wooden block of 2.54 cm on each side as used in the BBT [25]. Exclusion criteria were (1) a diagnosis of a brain injury other than stroke, (2) a diagnosed neglect, aphasia or hemianopsia and, (3) non-controlled medical conditions (chronic pain, drug abuse). All included patients were screened for dementia and stereopsis and their handedness was assessed. A Mini Mental State Examination (MMSE) of at least 20 points (light dementia or better) was required to participate in the study [26]. The stereopsis of each patient was evaluated with the Lang Stereotest 1 (LST), which shows three objects differing in disparity and perceived distance: a cat, a star and a car [27, 28]. It has a high predictive value for stereo-positivity in adults [28], which we assumed would be of importance in conducting the VPIT, which is represented in a 3D virtual environment on a computer screen. The study was approved by the Ethics Committee (KEK-Nr. 119/13) of the canton of Bern (Switzerland). All subjects gave their informed consent prior to study entry.

### Procedures

The measurements for the patients with stroke took place at the patients’ outpatient OT practice or in their home environment. The setting and test instructions for the BBT and NHPT corresponded to the standards set by Mathiowetz et al. (1985) [5, 6], translated into German by Schädler et al. (2011) [29]. The procedures followed for the VPIT were as described by Fluet et al. (2011) [17], with the following adaptations: 1) only 3 repetitions of the test were carried out as opposed to the 5 suggested by Fluet et al. (2011); 2) The force threshold to grasp and release pegs was set to 2 Newtons. This force threshold was empirically tested in our previous work with neurological patients, where it was shown to be adequate for most participants with mild to moderate hand impairment to perform the task [18, 19]; and 3) participants who needed assistance for the affected arm to perform the test were allowed to do so. The third adaptation was not initially planned, but proved necessary to implement after we observed major difficulties in performing the VPIT during the test trial in some participants (e.g., cursor alignment or regulation of the grasping force while holding the handle not precise enough). The participants were requested to first perform the BBT and the NHPT as per protocol. In some we noticed that participants needed support for the VPIT while the 2 clinical tests were already completed. It was too much due to fatigue to repeat them. The validity of this adaptation was addressed by a later subgroup analysis (see results section).

To minimize the effect of the required adaptation to a novel tool (VPIT), we gave patients a test trial prior to the measurements (on both days), where they were given all the time they needed to explore the virtual environment and get familiar to the functioning of the test. According to our previous work, this proved to be sufficient for patients to understand the task and the use of the VPIT.

All participants were tested twice with a 3–7 day interval between assessments. This time interval (mean 6.4 ± 0.5 days) was defined to minimize any learning effect that may occur from repeating the tests within a short time frame. During the first assessment session, demographic data were collected and the MMSE, FLANDERS and LST were carried out. Then, motor tests were performed in the following order of (1) BBT, (2) NHPT and (3) VPIT. For each test, one test trial (not timed) was allowed, which was then followed by three repetitions of the test. If needed, participants could have a rest between the motor test performances. All tests were done with the affected arm only where possible, with participants being allowed to use both hands for the VPIT as necessary. Therefore, depending on the result of the VPIT test trial (support of the affected arm needed/not needed), the following three repetitions of the VPIT were all performed accordingly. The second assessment session was composed of the test trial followed by three repetitions of the VPIT.

### Data analysis

SPSS version 22.0 (SPSS Inc, Chicago, Illinois) was used for data analysis. The study population and clinical characteristics were defined adopting descriptive statistics. The average of the 3 trials of each test was calculated and used for data analysis, as test-retest reliability is highest in all tests when the mean of three trials is used; lower correlations are known to occur when one trial or the highest score of three trials are utilized [30]. Normality of data was evaluated using the Shapiro Wilk test [31]. The level of statistical significance was set to *p* ≤ 0.05.

#### Validity

Concurrent validity was assessed by determining Spearman’s rank correlation coefficient (ρ) for the relationship between the VPIT/BBT and the VPIT/NHPT, respectively [31], namely for the parameters measuring a similar construct in both tests: (1) mean time in seconds (T_ex_[s]VPIT / T_ex_[s]NHPT), (2) mean number of dropped pegs (N_dp_VPIT / N_dp_NHPT), (3) mean number of dropped pegs/cubes (N_dp_VPIT / N_dc_BBT) and (4) mean number of transported cubes (T_ex_[s]VPIT / N_tc_BBT). The following correlation classification was used: no or very low: *ρ* = 0–0.25; low: *ρ* = 0.26–0.40; moderate: *ρ* = 0.41–0.69; high: *ρ* = 0.70–0.89; very high: *ρ* = 0.90–1.0 [32]. To measure the correlation between the mean number of dropped pegs/cubes during the VPIT/NHPT and VPIT/BBT, Cohen’s kappa was computed using GraphPad software (www.graphpad.com/quickcalcs) [33]. We hypothesized that (1) the correlations between T_ex_[s]VPIT / T_ex_[s]NHPT and T_ex_[s]VPIT / N_tc_BBT would be high for chronic patients with stroke (0.70–0.89). We further hypothesized that (2) the correlations between the number of pegs dropped during the VPIT and the number of pegs/cubes dropped during the conventional NHPT and BBT for chronic patients with stroke would be moderate (0.41–0.69).

#### Reliability

*Relative reliability* was determined by calculating intraclass correlation coefficients (ICCs) separately for average measures. In particular, we used the ICC 2 (A,k) formula (two-way mixed effects model where people effects are random and measures effects are fixed; k because the average of the three tests was used) [34, 35]. We selected the option “absolute agreement” in order to take into account the systematic error between raters, as there were two raters (BCT and JH) involved in data collection [36]. In this study, the same rater performed the test and the re-test procedure with a single participant. Furthermore, intensive pilot-testing was performed prior to data collection by both raters. The following classification was used: 0.90–0.99, high reliability; 0.80–0.89, good reliability; 0.70–0.79, fair reliability; 0.69 or below, poor reliability [37, 38]. We anticipated that the relative reliability of the 9 parameters measured with the VPIT would be good (ICC ≥ 0.80) [37].

To calculate *absolute reliability*, the ICCs were complemented by the Bland-Altman analysis, which can be used to show variation (or the magnitude of difference) of repeated measurements [39, 40]. The plots show the difference between test sessions 2 and 1 against the mean of the two test sessions for each subject [41, 42]. A free sample of the MedCalc statistical software version 14.8.1 (www.medcalc.org) was used to draw the Bland-Altman plots. The degree of heteroscedasticity was measured by calculating Kendall’s tau correlation (Τ) between the absolute differences and the corresponding means of each VPIT parameter. When a positive Τ > 0.1 was found, the data were considered heteroscedastic. When Τ < 0.1 or negative, the data were considered homoscedastic [43]. The data were logarithmically or square root transformed when heteroscedasticity was found [44, 45]. Thereafter, we calculated Kendall’s tau again; if Τ_trans_ decreased - indicating a more homoscedastic distribution of the data - reliability was analyzed using the transformed parameters [43].

To quantify the precision of individual scores on a test, we calculated the Standard Error of Measurement (SEM), using the formula $\mathrm{S}\mathrm{E}\mathrm{M}=\upsigma \sqrt{\left(1-\mathrm{I}\mathrm{C}\mathrm{C}\right)}$, with σ being the total variance of the scores from all subjects [34, 36]. We then calculated the Smallest Detectable Difference (SDD) based on the SEM, as follows: $\mathrm{S}\mathrm{D}\mathrm{D}=\mathrm{S}\mathrm{E}\mathrm{M}*1.96*\sqrt{2}$. As a last step we calculated: $\mathrm{S}\mathrm{D}\mathrm{D}\%=\frac{\mathrm{SDD}}{\mathrm{grand}\ \mathrm{mean}}*100$. The grand mean is the mean of the means of each VPIT parameter. As agreement parameters (SDDs) are expressed on the actual scale of the assessments, they allow clinical interpretation of the results [34, 36]. Furthermore, the SDD% can be used to compare test-retest reliability among tests [25]. We hypothesized that the SDD% is ≤ 54 % of the mean average values of the VPIT, as Chen et al. (2009) found an SDD% of 54 % for the affected hand using the NHPT in patients with stroke [25].

## Results

The demographic and clinical characteristics of the participants are summarized in Table 1 and the results of the achieved scores (BBT, NHPT, VPIT) in Table 2. From the 33 chronic patients with stroke initially recruited, two participants dropped out: one only completed 2 of the 3 required VPIT test trials due to poor physical health, whereas the second could not perform the VPIT task with the affected hand. In 26 participants (83.9 %), stroke had occurred for the first time, while 5 (16.1 %, 4 men and 1 woman) had suffered at least two stroke events. Although all participants fulfilled the inclusion criteria, 11 patients were able to perform all motor tests solely with the affected arm, whereas 20 patients also required support from the non-affected arm to perform the VPIT, as they possibly were fatigued by the duration of the tests. This support was needed to increase stability of the affected arm and therefore motor control of the affected hand. To account for these differences in test performance (with and without support), we decided to additionally conduct a subgroup analysis, subgroup 1 (*n* = 11) being without support of the non-affected arm and subgroup 2 (*n* = 20): being those who required support. Shapiro Wilk testing indicated too much difference within the validity and test-retest reliability data to be normally distributed.

**Table 1:** Demographics and clinical characteristics of participants (*n* = 31)

Characteristic	Value
Sex, *n* (%)	
female	8 (25.8)
male	23 (74.2)
Age, in years	
mean ± SD	62.7 ± 15.1
Time onset stroke, in months	
mean ± SD	51.1 ± 82.0
Affected hand, *n* (%)	
right	17 (54.8)
left	14 (45.2)
Learned left-handedness due to stroke, *n* (%)	7 (22.6)
Affected hand = dominant hand, *n* (%)	18 (58.1)
LST, recognized images, *n* (%)	
3 (out of 3)	5 (16.1)
2 (out of 3)	12 (38.7)
1 (out of 3)	11 (35.5)
0 (out of 3)	3 (9.7)
MMSE	
mean ± SD	27.5 ± 2.4

*LST* Lang Stereotest 1, *MMSE* Mini Mental Status Examination, *n* number, *SD* Standard Deviation

**Table 2:** Achieved scores of the VPIT, NHPT and BBT

Parameters	Mean ± SD of achieved scores		
mean of 3 trials	All patients (*n* = 31)	Subgroup 1: without support (*n* = 11)	Subgroup 2: with support (*n* = 20)
T_ex_ [s] VPIT	119.7 ± 72.1	113.1 ± 64.5	123.3 ± 77.2
T_ex_ [s] NHPT	54.4 ± 28.9	32.2 ± 15.3	66.7 ± 27.5
T_ex_ [s] NHPT_(TP1)_	39.4 ± 20.5	22.5 ± 10.5	48.6 ± 18.7
N_tc_ BBT	35.8 ± 17.4	51.4 ± 13.8	27.2 ± 12.6
N_dp_ VPIT	0.5 ± 1.1	0.1 ± 0.2	0.8 ± 1.3
N_dp_ NHPT	0.8 ± 0.9	0.4 ± 0.4	1.0 ± 1.0
N_dc_ BBT	0.7 ± 0.9	0.7 ± 0.8	0.7 ± 0.9

*T*_*ex*_ *[s]* execution time in seconds, *VPIT* Virtual Peg Insertion Test, *NHPT* Nine Hole Peg Test, *TP1* time point 1 (time stopped at the point when all 9 pegs were put in the 9 holes (without replacing them in the dish)), *BBT* Box and Block Test, *N*_*tc*_ number of transported cubes, *N*_*dp*_ number of dropped pegs, *N*_*dc*_ number of dropped cubes, *SD* Standard Deviation

### Concurrent validity

The results of the concurrent validity calculations (ranges) are presented in Table 3. The correlations between the VPIT and BBT/NHPT were low (*ρ* = −0.23–0.31) and non-significant (*p* = 0.09–0.51) in all parameters. The correlations between the VPIT and BBT/NHPT for subgroup 1 were moderate (*ρ* = −0.41–0.61) and non-significant (*p* = 0.07–0.60). The correlations between the VPIT and BBT/NHPT for subgroup 2 were low (*ρ* = −0.21–0.35) and non-significant (*p* = 0.13–0.51). The strength of agreement for N_dp_VPIT / N_dp_NHPT and no (zero) dropped pegs in the VPIT and NHPT was considered to be poor (Kappa = 0.041; SE of kappa = 0.176; 95 % CI = −0.30–0.39) with 16 (51.6 %) observed agreements. Accordingly, the kappa agreement for the N_dp_VPIT / N_dc_BBT and no (zero) dropped pegs/cubes during those tests was poor (Kappa = 0.189; SE of kappa = 0.157; 95 % CI = −0.12–0.50) with 18 (58.1 %) observed agreements.

**Table 3:** Concurrent validity of the VPIT with the NHPT and the BBT

Concurrent validity parameters	Spearman’s rank correlation coefficient		
mean of 3 trials	All patients	Subgroup 1: without support	Subgroup 2: with support
	(*n* = 31)	(*n* = 11)	(*n* = 20)
T_ex_ [s] VPIT /	0.31	0.57	0.35
T_ex_ [s] NHPT	0.09*	0.07*	0.13*
T_ex_ [s] VPIT /	0.30	0.61	0.32
T_ex_ [s] NHPT_(TP1)_	0.10*	0.47*	0.16*
T_ex_ [s] VPIT /	−0.23	−0.41	−0.21
N_tc_ BBT	0.22*	0.22*	0.37*
N_dp_ VPIT /	0.21	−0.18	0.17
N_dp_ NHPT	0.26*	0.60*	0.49*
N_dp_ VPIT /	−0.12	−0.18	−0.16
N_dc_ BBT	0.51*	0.60*	0.51*

*p-values: level of significance: *p* ≤ 0.05

*T*_*ex*_ *[s]* execution time in seconds, *VPIT* Virtual Peg Insertion Test, *NHPT* Nine Hole Peg Test, *TP1* time point 1 (time stopped at the point when all 9 pegs were put in the 9 holes (without replacing them in the dish)), *BBT* Box and Block Test, *N*_*tc*_ number of transported cubes, *N*_*dp*_ number of dropped pegs, *N*_*dc*_ number of dropped cubes, *SD* Standard Deviation

### Test-retest reliability

**Fig. 2:**
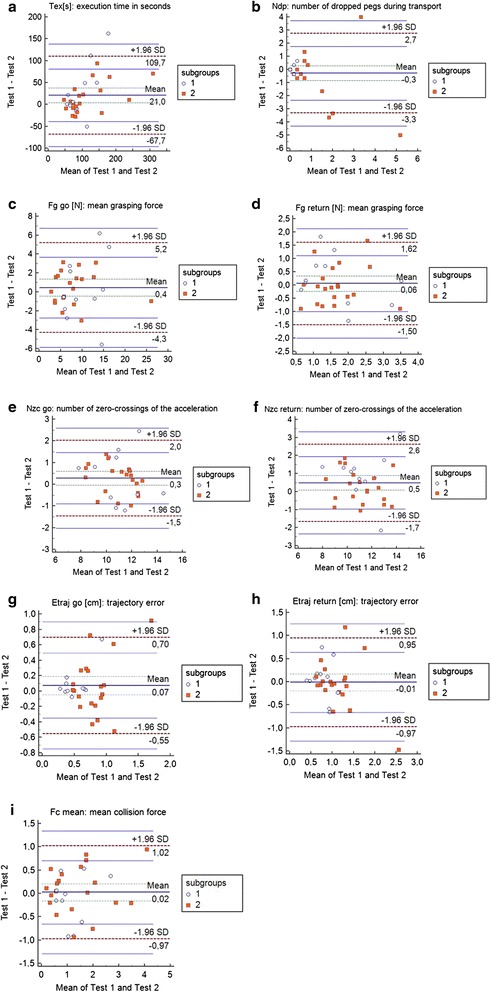
Bland-Altman plots of the 9 VPIT parameters. Plotted differences of (**a**) T_ex_[s]: execution time in seconds, (**b**) N_dp_: number of dropped pegs during transport, (**c**) F_g_go[N]: mean grasping force, (**d**) F_g_return[N]: mean grasping force, (**e**) Nzc[1/s]go/return: number of zero-crossings of the acceleration, (**f**) Nzc[1/s]go/return: number of zero-crossings of the acceleration, (**g**) E_traj_go[cm]: trajectory error, (**h**) E_traj_return[cm]: trajectory error, (**i**) F_cmean_[N]: mean collision force. ○ Represents subgroup 1 (*n* = 11); (orange square) Represents subgroup 2 (*n* = 20) (red broken line) Lines for 95 % CI of limits of agreement, (green broken line) Lines for 95 % CI of mean of differences

The 9 test-retest reliability parameters of the VPIT are presented in Table 4 for the whole study population and in Table 5 (subgroup 1) and 6 (subgroup 2) for the subgroups. All VPIT parameters are illustrated in Fig. 2 by Bland-Altman plots. For the whole stroke sample, the correlations for the 5 parameters T_ex_[s], F_g_go[N], Nzc[1/s]go/return and F_cmean_[N] were good to high (ICCs = 0.83–0.94, SEMs = 0.07–0.63, except for the T_ex_[s] with SEM = 25.34). Fair reliability was found for the parameters F_g_return (ICC = 0.75, SEM = 0.43) and E_traj_go[cm] (ICC = 0.70, SEM = 0.19). Poor reliability was measured for E_traj_return[cm] (ICC = 0.67, SEM = 0.29) and N_dp_ (ICC = 0.58, SEM = 0.14). The SDD were ≤ 54 % in all VPIT parameters (SDD% = 1.37–21.42) except for T_ex_[s] with SDD% = 434.5 %.

**Table 4:** Test-retest reliability parameters of the whole stroke sample (*n* = 31)

VPIT parameters	Test	Retest	Test-Retest	Kendall’s tau correlation	Kendall’s tau correlation with transformed data	ICC (95 % CI)	SEM agreement	SDD	SDD%
	Mean ± SD	Mean ± SD	Mean difference ± SD						
T_ex_ [s]	119.71 ± 72.05	98.71 ± 50.77	21.01 ± 45.25	0.01	-	0.83 (0.61–0.92)	25.34	70.18	434.50
N_dp_	0.54 ± 1.06	0.83 ± 1.59	−0.29 ± 1.55	0.16^a^	0.12^b,c^	0.51 (−0.01–0.76)	0.95	2.62	16.22
	0.13 ± 0.19^c^	0.18 ± 0.23^c^				0.58 (0.13–0.79)^c^	0.14^c^	0.38^c^	2.64^c^
F_g_ go [N]	8.98 ± 5.15	8.54 ± 4.96	0.44 ± 2.43	0.10	-	0.94 (0.87–0.97)	0.14	3.46	21.42
F_g_ return [N]	1.66 ± 0.83	1.60 ± 0.91	0.06 ± 0.80	0.08	-	0.75 (0.47–0.88)	0.43	1.18	7.31
N_zc [1/S]_ go	11.09 ± 1.52	10.80 ± 1.70	0.28 ± 0.89	0.21^a^	0.20^b,d^	0.91 (0.82–0.96)	0.48	1.33	8.23
	3.32 ± 0.23^d^	3.28 ± 0.26^d^				0.92 (0.82–0.96)^d^	0.07^d^	0.20^d^	1.37^d^
N_zc [1/s]_ return	11.36 ± 1.69	10.88 ± 1.76	0.48 ± 1.09	0.14^a^	0.20^c^	0.87 (0.71–0.94)	0.63	1.75	10.84
E_traj_ go [cm]	0.74 ± 0.38	0.67 ± 0.29	0.07 ± 0.32	0.03	-	0.70 (0.39–0.85)	0.19	0.53	3.28
E_traj_ return [cm]	0.99 ± 0.40	1.00 ± 0.56	0.01 ± 0.49	0.18^a^	0.20^c^	0.67 (0.30–0.84)	0.29	0.80	4.95
F_cmean_ [N]	1.32 ± 1.03	1.29 ± 0.95	0.02 ± 0.51	0.06	-	0.93 (0.86–0.97)	0.26	0.73	4.52

^a^Τ > 0.1, T_trans_ decreased^b^, ^c^ = log10 transformed, ^d^ = square root transformed

*T*_*ex*_ *[s]* execution time in seconds, *N*_*dp*_ number of dropped pegs during transport, *F*_*g*_ *(go/return) [N]* mean grasping force of the three force sensors integrated into the handle in Newton, *Nzc[1/s](go/return)* number of zero-crossings of the acceleration, *E*_*traj*_ *[cm]* trajectory error, *F*_*cmean*_ mean collision force, *SD* Standard Deviation, *ICC* Intraclass Correlation Coefficient, *CI* Confidence Interval, *SEM* Standard Error of Measurement, *SDD* Smallest Detectable Difference

**Table 5:** Test-retest reliability parameters for subgroup 1 (*n* = 11) without support of the non-affected arm

	Subgroup 1 (*n* = 11) VPIT test performance without support								
VPIT parameters	Test Mean ± SD	Retest Mean ± SD	Test-Retest Mean difference ± SD	Kendall’s tau correlation	Kendall’s tau correlation with transformed data	ICC (95 % CI)	SEM agreement	SDD	SDD%
T_ex_ [s]	113.14 ± 64.54	83.56 ± 27.26	29.57 ± 61.17	.19^a^	0.31^c^	0.35 (−0.90–0.81)	37.01	102.50	682.87
N_dp_	0.12 ± 0.23	0.12 ± 0.23	0.0 ± 0.34	0.0	-	0.25 (−6.04–0.69)	0.20	0.55	3.66
F_g_ go [N]	10.49 ± 4.84	10.13 ± 4.09	0.36 ± 3.4	0.20^a^	0.24^c^	0.84 (0.40–0.96)	1.79	4.95	32.97
F_g_ return [N]	1.74 ± 0.92	1.55 ± 1.22	0.19 ± 0.93	0.13^a^	0.13^d^	0.78 (0.18–0.94)	0.50	1.39	9.26
N_zc [1/s]_ go	11.18 ± 1.79	10.79 ± 1.97	0.39 ± 1.21	0.06	-	0.89 (0.60–0.97)	0.62	1.73	11.53
N_zc [1/s]_ return	11.72 ± 1.91	10.80 ± 1.83	0.92 ± 1.29	0.09	-	0.82 (0.28–0.95)	0.79	2.20	14.66
E_traj_ go [cm]	0.56 ± 0.27	0.47 ± 0.15	0.09 ± 0.20	0.16^a^	0.10^b,c^	0.70 (0.00–0.92)	0.12	0.32	2.13
	0.29 ± 0.18^c^	0.35 ± 0.15^c^	0.06 ± 0.11^c^			0.83 (0.38–0.95)^c^	0.07^c^	0.19^c^	1.27^c^
E_traj_ return [cm]	0.79 ± 0.28	0.75 ± 0.35	0.04 ± 0.42	0.06	-	0.18 (−2.80–0.80)	0.29	0.79	5.26
F_cmean_ [N]	1.08 ± 0.72	1.18 ± 0.66	−0.10 ± 0.52	0.02	-	0.84 (0.41–0.96)	0.28	0.77	5.13

^a^Τ > 0.1, T_trans_ decreased^b^, ^c^ = log10 transformed, ^d^ = square root transformed

*T*_*ex*_ *[s]* execution time in seconds, *N*_*dp*_ number of dropped pegs during transport, *F*_*g*_ *(go/return) [N]* mean grasping force of the three force sensors integrated into the handle in Newton, *N*_*zc [1/s]*_ *(go/return)* number of zero-crossings of the acceleration, *E*_*traj*_ *[cm]*: trajectory error, *F*_*cmean [N]*_ mean collision force, *SD* Standard Deviation, *ICC* Intraclass Correlation Coefficient, *CI* Confidence Interval, *SEM* Standard Error of Measurement, *SDD* Smallest Detectable Difference

The subgroup analysis of subgroup 1 (*n* = 11) showed good ICCs for the 5 VPIT parameters F_g_go[N], Nzc[1/s]go/return, E_traj_go and F_cmean_ (ICCs = 0.82–0.89, SEMs = 0.07–1.79). Fair reliability was found in the parameter F_g_return[N] (ICC = 0.78, SEM = 0.50), while T_ex_[s], N_dp_ and E_traj_return[cm] showed poor reliability (ICCs = 0.18–0.35 with SEMs = 0.20–37.01). All VPIT parameters showed SDD of ≤ 54 % (SDD% = 1.27–32.97) except for T_ex_[s] with SDD% = 682.87 %.

Subgroup 2 (*n* = 20) showed high ICCs in 5 out of 9 VPIT parameters (T_ex_[s], F_g_go[N], Nzc[1/s]go/return and F_cmean_[N] with ICCs = 0.92–0.97, SEMs = 0.02–19.25). Fair reliability was found in F_g_return[N] (ICC = 0.73, SEM = 0.40). The remaining 3 parameters (N_dp_ and E_traj_go/return[cm]) showed poor reliability (ICCs = 0.49–0.69, SEMs = 0.10–0.22). Within subgroup 2, all VPIT parameters showed SDD% values of ≤ 54 % (SDD% = 0.26–15.08 %) except for T_ex_[s] with SDD% = 317.76 %.

## Discussion

This is the first study evaluating the test-retest reliability of the novel VPIT and its concurrent validity with conventional upper limb function tests in chronic patients with stroke. In this stroke sample (*n* = 31), the VPIT presented seven out of nine (78 %) reliable parameters that passed the accepted minimal standards for group comparisons (ICC ≥ 0.70) with ICCs = 0.70–0.94 [46]. The SDD% values were small in all VPIT parameters (1.37–21.42 %) except for T_ex_[s] (SDD% = 434.5 %). The correlations of the execution time and the number of dropped pegs/cubes of the VPIT with the NHPT and the BBT, respectively, were (ρ = −0.23–0.31), although non-significant (*p* = 0.09–0.51).

### Concurrent validity

For the concurrent validity part of this study, both hypotheses were rejected, as the correlations of the VPIT with the conventional tests NHPT and BBT were low. The rejection of *hypothesis 1* (correlations for the T_ex_[s]VPIT / T_ex_[s]NHPT and T_ex_[s]VPIT / N_tc_BBT) might be due to the following reasons: **(1)** the diverse and unstable upper limb skills of the study sample led to a large inter-subjects variation, which can be seen in the high Standard Deviation (SD) of T_ex_[s]VPIT with 72.1 s and SD = 28.9 s for the NHPT, respectively (Table 2). Moreover, support of the affected-arm by the non-affected arm might have caused further variability in the data, **(2)** Although the test performance of the NHPT was stable within the 3 test trials (mean SD (average from each subject’s SD) = 5.2 s), there was variation within the 3 test trials of the VPIT (mean SD = 27.2 s). The higher intra-subject variation in the T_ex_[s]VPIT parameter could be attributed to the nature of the VPIT, which is not a real physical object manipulation test and involves tools that patients are not familiar with (e.g., the robotic handle and the computer for some patients). This is supported by Bowler et al. (2011), who achieved a more accurate and consistent data set for an embedded NHPT than for a purely hapto-VR-NHPT version [47]. As the VPIT was the last assessment administered in session one, it might be that patients were already more tired and less able to concentrate than in the NHPT. This observation is supported by the smaller SD of the VPIT during the second assessment session (see Table 4). Furthermore, compared to the BBT, the T_ex_[s]VPIT has no time limit, thus the participants with poorer upper limb function had to perform the assessment even longer than the more skilled ones, also resulting in increased fatigue of the affected arm from test trial to test trial (although they were allowed to have a rest between tests). Conversely, the correlations of the VPIT with the NHPT - the two tests with no time limit - were better than those with the BBT (time limit: 60 s). A reason for this could be the similar test procedures of the VPIT / NHPT, whereas the BBT has different test procedures [5].

The rejection of *hypothesis 2* (correlations for N_dp_VPIT with the N_dp_NHPT / N_dc_BBT) occurred due to very low correlations between those validity parameters. The scatter plots of those parameters showed no linear correlation, as many participants did not drop any pegs or cubes during test trials (no drops *n* = 17 in VPIT (55 %); *n* = 10 in NHPT (32 %) and *n* = 14 in BBT (45 %)). This ceiling-effect - considered to be present if more than 15 % of all participants received the highest possible score (here: dropping no peg/cube) [48] - did not allow a distinction from the participants with the highest achievable score, indicating limited validity. This finding is supported by the poor strength of agreement for the N_dp_ and no dropped pegs in the VPIT and NHPT (Kappa = 0.04) or BBT (Kappa = 0.19), respectively. Furthermore, the high SDs of the N_dp/c_ (VPIT/BBT/NHPT) parameters indicate the high variance of the study sample (Table 2). The high variance in the VPIT could be due to the difficulty of the coordination of the PHANTOM Omni arm and the virtual pegboard in the virtual 3D space, especially for subgroup 2 (those with the higher N_dp_ parameter than subgroup 1; see Table 2). Most participants in this group had difficulty in aligning the handle of the haptic display to the dropped peg lying on the virtual board, which resulted in several grasp attempts and the associated higher drop rate of pegs.

Furthermore, the VPIT handle remains in the patients hand during the entire test, but inserting a peg in the hole requires control of grasping force and to decrease applied grasping force below the 2 N force threshold. Also, patients have to be below the 2 N threshold before being able to grasp a new peg (i.e., the patient cannot just tightly grasp the handle during the whole test and “only” align cursor to pegs/holes to achieve the task, see Fluet et al. (2011) [17] and the force traces presented in that paper may illustrate how force control (grasping AND releasing) is required for achieving the task. Nevertheless, this may have influenced the performance time of the VIPT and also the low values for concurrent validity. The degree of stereopositivity (Table 1), which we assumed to be important to perform the VPIT [28], didn’t seem to be indicative of the participants being able to perform the VPIT or not, as all participants could perform it equally. However, further investigations into this observation would be necessary to allow us to draw a comprehensive conclusion.

### Test-retest reliability

In the hypothesis concerning the *relative test-retest reliability*, we expected the ICCs to be ≥ 0.80. This assumption was met by 5 out of 9 VPIT parameters in the whole stroke sample (Table 4) and both subgroups (Tables 5 and 6). Those 5 parameters were the same for the whole sample and for subgroup 2 (T_ex_[s], F_g_go[N], N_zc[1/s]_go/return and F_cmean_[N]), with slightly higher correlations in subgroup 2 than for the whole sample. This is not surprising, as subgroup 1 with the highest mean differences between test and retest (Table 5) is not included, indicating that supporting the affected arm allowed a more stable test performance in subgroup 2. The lower ICCs in subgroup 1 might also be due to the lower number of participants (*n* = 11) [49], as well as due to a learning effect in some parameters; e.g., the important decrease in T_ex_[s] of 29.6 s from test to retest (Table 5). This finding is illustrated by the Bland-Altman plot (Fig. 2a)), where 4 subjects in subgroup 1 improved by > 50 s.

**Table 6:** Test-retest reliability parameters for subgroup 2 (*n* = 20) with support of the non-affected arm

	Subgroup 2 (*n* = 20) VPIT test performance with support								
VPIT parameters	Test Mean ± SD	Retest Mean ± SD	Test-Retest Mean difference ± SD	Kendall’s tau correlation	Kendall’s tau correlation with transformed data	ICC (95 % CI)	SEM agreement	SDD	SDD%
T_ex_ [s]	123.33 ± 77.24	107.04 ± 58.93	16.30 ± 34.62	0.03	-	0.92 (0.79–0.97)	19.25	53.32	317.76
N_dp_	0.77 ± 1.27	1.22 ± 1.87	−0.45 ± 1.91	0.18^a^	0.11^b,c^	0.45 (−0.37–0.78)	1.16	3.23	19.25
	0.18 ± 0.22^c^	0.26 ± 0.26^c^	−0.07 ± 0.28^c^			0.49 (−0.26–0.80)^c^	0.17^c^	0.48^c^	3.11^c^
F_g_ go [N]	8.15 ± 5.25	7.67 ± 5.28	0.49 ± 1.79	0.11^a^	0.12^d^	0.97 (0.93–0.99)	0.91	2.53	15.08
F_g_ return [N]	1.62 ± 0.80	1.63 ± 0.73	−0.02 ± 0.72	0.10^a^	-	0.73 (0.29–0.89)	0.40	1.10	6.56
N_zc [1/s]_go	11.03 ± 1.39	10.81 ± 1.59	0.22 ± 0.68	0.34^a^	0.32^b,c^	0.94 (0.86–0.98)	0.37	1.01	6.02
	1.04 ± 0.06^c^	1.03 ± 0.07^c^	0.01 ± 0.03^c^			0.94 (0.85–0.98)^c^	0.02^c^	0.04^c^	0.26^c^
N_zc[1/s]_ return	11.17 ± 1.57	10.93 ± 1.77	0.25 ± 0.91	0.18^a^	0.18^d^	0.92 (0.80–0.97)	0.47	1.31	7.81
E_traj_ go [cm]	0.84 ± 0.39	0.78 ± 0.28	0.06 ± 0.37	0.01	-	0.58 (−0.06–0.84)	0.22	0.60	3.58
E_traj_ return [cm]	1.10 ± 0.42	1.15 ± 0.61	−0.04 ± 0.53	0.16^a^	0.15^b,c^	0.66 (0.13–0.87)	0.30	0.83	4.95
	0.02 ± 0.16^c^	0.02 ± 0.19^c^	0.00 ± 0.17^c^			0.69 (0.21–0.88)^c^	0.10^c^	0.27^c^	1.75^c^
F_cmean_ [N]	1.45 ± 1.16	1.36 ± 1.08	0.09 ± 0.50	0.10^a^	0.01^b,d^	0.95 (0.87–0.98)	0.25	0.69	4.11
	1.11 ± 0.47^d^	1.08 ± 0.47^d^	0.04 ± 0.23^d^			0.94 (0.84–0.98)^d^	0.12^d^	0.32^d^	2.07^d^

^a^Τ > 0.1, T_trans_ decreased^b^, ^c^ = log10 transformed, ^d^ = square root transformed

*T*_*ex*_ *[s]* execution time in seconds, *N*_*dp*_ number of dropped pegs during transport, *F*_*g*_ *(go/return) [N]* mean grasping force of the three force sensors integrated into the handle in Newton, *N*_*zc[1/s]*_ *(go/return)* number of zero-crossings of the acceleration, *E*_*traj*_ *[cm]* trajectory error, *F*_*cmean [N]*_ mean collision force; *SD* Standard Deviation, *ICC* Intraclass Correlation Coefficient, *CI* Confidence Interval, *SEM* Standard Error of Measurement, *SDD* Smallest Detectable Difference

The kinetic parameters F_g_go[N] and F_cmean_[N] and the kinematic parameters Nzc[1/s]go/return achieved high test-retest reliability in the whole sample and in both subgroups. This means that the stroke participants were able to transport the pegs with a constant grip strength and movement coordination while transporting the peg (go). However, there were fair correlations when approaching a new peg (return) (F_g_return) in the whole sample and both subgroups. This might be due to the fact that participants still had to hold an object (the handle) on the way back to approach a new peg without actually carrying a peg. Furthermore, if we look at the overall test-retest reliability of the whole study sample for N_dp_, it was poor (ICC = 0.58). This might be due to the artificial force threshold to grasp and release the pegs, which is quite unintuitive, as there is no feedback provided on the force applied by the subject and on the force threshold, although measured by the handle (F_g_go/return[N]). This might lead many subjects to drop the pegs. However, to achieve a conclusive statement regarding the clinical use of those unique kinetic and kinematic VPIT parameters, further research is needed. This could be done by comparing the reliability of stroke results with healthy controls, or by evaluating their validity with other outcome measurements quantifying force control and movement coordination.

From the 5 VPIT parameters meeting the hypothesis for *relative test-retest reliability*, all VPIT parameters fulfilled the hypothesis for the absolute test-retest reliability with SDD% of ≤ 54 % (Tables 4, 5 and 6) except for the parameter T_ex_[s]. This is quite surprising, as more complex and demanding upper limb function tests – such as the VPIT – have in general higher SDDs than simpler tests – such as the BBT or NHPT. Therefore, our results are in contrast with those of Chen et al. (2009) [25], whose SDD% values were high for the affected hand, especially in the NHPT, with an SDD% of 52 % for the nonspastic and 88 % for the spastic group. In our sample however, the only VPIT parameter not being susceptible to change was T_ex_[s], as its SDD was high and varied greatly within the whole sample and both subgroups (SDDs = 53.3–102.5 s). In other words, only a change between two consecutive measurements exceeding at least 53 s for T_ex_[s] can be interpreted as a true clinical improvement when chronic patients with stroke perform the test with the affected hand. In addition, the SDD can be used as a threshold to identify statistically significant individual change [50, 51]. Thus, if a change between 2 consecutive measurements for an individual patient exceeds the SDD (e.g., 53.3 s in subgroup 2) the individual patient may be exhibiting significant improvement. In our sample, this was the case for 4 participants for the T_ex_[s] parameter in subgroup 2, while one participant exceeded 102.5 s in subgroup 1. The data show that allowing support of the affected arm increased performance stability in the test and retest. This can be seen in the almost twice as high SDD of subgroup 1 (SDD = 102.5 s) compared to subgroup 2 with an SDD of 53.3 s.

### Limitations and future research

As the VPIT extracts much more (i.e., kinetic and kinematic) parameters than conventional, non-VR upper limb function assessments (i.e., time, number of dropped/transported objects), it was not possible to validate all of them. Therefore, future studies should focus on the validity of those parameters measurable by VR devices by comparing them with other VR-based upper limb function assessments. Furthermore, it would be of interest to evaluate the discriminant validity of the VPIT by comparing stroke participants’ performances with those of healthy controls.

Future versions of the VPIT should provide visual feedback on the force applied by the subject and on the force threshold. Another limitation might be the fact that two raters collected data, although the test-retest measurements of one participant were performed by the same rater. However, the major limitation of our study can be seen in the given permission to support the weight of the affected arm with the non-affected arm (and not use the other hand to steer), visually monitored by the supervising rater (occupational therapist). We are aware that this can be judged as a bias, because the test performance was not identical between the two subgroups, nor can we be sure how much the non-affected arm really supported (steered) the affected arm. Nevertheless, allowing support of the affected arm opens the use of the VPIT for a motorically weaker stroke population. If available, adjustable armrests (e.g., the Armon Elemento [52]) could be used instead of the non-affected arm, which might improve body stability and therefore the test performance by providing repeatable conditions from trial to trial and patient to patient. Randomisation of the several tests (NHPT, BBT, VPIT) may also reduce tiredness of the patients and increase validity.

Last, the sample size was relatively small and may have affected the values of the reproducibility and measurement error. A sample size of at least 50 is generally seen as adequate for the assessment of the agreement parameter, based on a general guideline by Altman [53]. The sample size we used of 33 patients with chronic stroke is, however, a realistic group size to find first estimates for the assumed relation between stroke and the hemi paretic arm / hand for the VPIT. Future studies may therefore strive for a bigger study sample and evaluate responsiveness to treatment of the VPIT.

### Implications for practice

The VPIT provides more objective and comprehensive measurements of upper limb function than conventional, non-computerized hand assessments. Receiving feedback concerning for example the patient’s ability to control force or movement coordination is essential for rehabilitation. Furthermore, getting information about movement smoothness, as provided by the VPIT, offers clinically relevant information, as smoothness has been shown to be a good indicator of upper limb coordination and stroke recovery [23]. Therefore, clinicians can use these information to work on those motor skills crucial for the independent performance of daily activities [54]. Thanks to its compact and transportable shape, the VPIT is easy to administer even in the patient’s home, thus allowing a broad use amongst clinicians working in different settings. As all test results are stored in the computer and could be graphically displayed immediately after completion of the test, performance can be discussed with the patient and adjustments made to the therapy program of a patient depending on the progress. To increase the ease of use of the VPIT, time limitations in the test duration for motorically weaker patients should be considered, together with the allowance to use adjustable armrests if needed.

An important aim of developing this new assessment is to improve the assessment of arm function in a clinical setting where the results of the assessment can be generalized to a population reflective of the “real world”. Seen from this perspective it is rather a strong point of our study that we had a rather heterogeneous sample because this can be considered more realistic for clinical settings.

## Conclusions

The VPIT is a promising upper limb function assessment which has proved to be feasible for use with this diverse group of chronic patients with stroke. The low concurrent validity showed that the VPIT was inherently different from the conventional tasks, indicating that performing this hapto-virtual reality assessment requires other components of upper limb motor performance than the NHPT and BBT. The high test-retest reliability in 5 and the low SDD% in 8 out of 9 VPIT parameters showed that those parameters remain consistent when performed by patients with chronic stroke and are susceptible to change, allowing diagnostic and therapeutic use in clinical practice for this patient group. The other 4 parameters (N_dp_, F_g_return[N] and E_traj_go/return[cm]) showed poor to fair ICCs when performed with the affected hand and require further research for this population. The high intra-subject variation indicated that the VPIT is a demanding test for this stroke sample, which requires a thorough introduction to this assessment. Allowing testing trials before starting with the assessment is a prerequisite for a reliable test performance. When using the VPIT as outcome measurement, clinicians may want to use the SDDs reported in this article as reference points for clinically important changes, and the SDD% results to compare test-retest reliability among other tests.

## Abbreviations

BBT: Box and Block Test
CI: Confidence Interval
E_traj_[cm]: Trajectory error in centimeters
ICC: Intraclass Correlation Coefficient
F_cmean_: Mean collsion force
F_g_[N]: Mean grasping force in Newton
N_dc_: Number of dropped cubes
N_dp_: Number of dropped pegs
NHPT: Nine Hole Peg Test
N_tc_: Number of transported cubes
N_zc_Nzc[_1/s_]: Number of zero-crossings
SD: Standard Deviation
SDD: Smallest Detectable Difference
SEM: Standard Error of Measurement
T_ex_[s]: Execution time in seconds
VPIT: Virtual Peg Insertion Test

## Footnotes

1 104W13400 Donges Bay Road, PO Box 1005, Germantown, Wisconsin 53022-8205

## References

[1] Lundborg G (2014). The Hand and the Brain. From Lucy’s thumb to the tought-controlled robotic hand.

[2] Johansson BB (2011). Current trends in stroke rehabilitation. A review with focus on brain plasticity. Acta Neurol Scand.

[3] Fluet GG, Deutsch JE (2013). Virtual reality for sensorimotor rehabilitation post-stroke: the promise and current state of the field. Curr Phys Med Rehabil Rep.

[4] Lin KC, Chuang LL, Wu CY, Hsieh YW, Chang WY (2010). Responsiveness and validity of three dexterous function measures in stroke rehabilitation. J Rehabil Res Dev.

[5] Mathiowetz V, Volland G, Kashman N, Weber K (1985). Adult norms for the Box and Block Test of manual dexterity. Am J Occup Ther.

[6] Mathiowetz V, Weber K, Kashman N, Volland G (1985). Adult norms for the Nine Hole Peg Test of finger dexterity. Occup Ther J Res.

[7] Oxford Grice K, Vogel KA, Le V, Mitchell A, Muniz S, Vollmer MA (2003). Adult norms for a commercially available Nine Hole Peg Test for finger dexterity. Am J Occup Ther.

[8] Laver KE, George S, Thomas S, Deutsch JE, Crotty M (2011). Virtual reality for stroke rehabilitation. Cochrane Database Syst Rev.

[9] Brewer L, Horgan F, Hickey A, Williams D (2013). Stroke rehabilitation: recent advances and future therapies. QJM.

[10] Yeh SC, Lee SH, Chan RC, Chen S, Rizzo A (2014). A virtual reality system integrated with robot-assisted haptics to simulate pinch-grip task: Motor ingredients for the assessment in chronic stroke. NeuroRehabilitation.

[11] Fordell H, Bodin K, Bucht G, Malm J (2011). A virtual reality test battery for assessment and screening of spatial neglect. Acta Neurol Scand.

[12] Lee JH, Ku J, Cho W, Hahn WY, Kim IY, Lee SM (2003). A virtual reality system for the assessment and rehabilitation of the activities of daily living. Cyberpsychol Behav.

[13] Amirabdollahian F, Johnson G (2011). Analysis of the results from use of haptic peg-in-hole task for assessment in neurorehabilitation. Appl Bionics Biomechanics.

[14] Bardorfer A, Munih M, Zupan A, Primozic A (2001). Upper limb motion analysis using haptic interface. IEEE ASME Trans Mechatron.

[15] Feys P, Alders G, Gijbels D, De Boeck J, De Weyer T, Coninx K (2009). Arm training in multiple sclerosis using phantom: Clinical relevance of robotic outcome measures. IEEE International Conference on Rehabilitation Robotics.

[16] Xydas E, Louca L (2009). Upper limb assessment of people with multiple sclerosis with the use of a haptic nine hole peg-board test. Proceedings of the 9th biennal Conference on Engineering Systems Design and Analysis.

[17] Fluet M-C, Lambercy O, Gassert R (2011). Upper limb assessment using a virtual peg insertion test. Proc IEEE International Conference on Rehabilitation Robotics (ICORR); Switzerland, Zurich.

[18] Lambercy O, Fluet MC, Lamers I, Kerkhofs L, Feys P, Gassert R (2013). Assessment of upper limb motor function in patients with multiple sclerosis using the Virtual Peg Insertion Test: a pilot study. IEEE Int Conf Rehabil Robot.

[19] Gagnon C, Lavoie C, Lessard I, Mathieu J, Brais B, Bouchard JP (2014). The Virtual Peg Insertion Test as an assessment of upper limb coordination in ARSACS patients: a pilot study. J Neurol Sci.

[20] Nordin N, Xie SQ, Wunsche B (2014). Assessment of movement quality in robot- assisted upper limb rehabilitation after stroke: a review. J Neuroeng Rehabil.

[21] Kim H, Miller LM, Fedulow I, Simkins M, Abrams GM, Byl N (2013). Kinematic data analysis for post-stroke patients following bilateral versus unilateral rehabilitation with an upper limb wearable robotic system. IEEE Trans Neural Syst Rehabil Eng.

[22] Panarese A, Colombo R, Sterpi I, Pisano F, Micera S (2012). Tracking motor improvement at the subtask level during robot-aided neurorehabilitation of stroke patients. Neurorehabil Neural Repair.

[23] Rohrer B, Fasoli S, Krebs HI, Hughes R, Volpe B, Frontera WR (2002). Movement smoothness changes during stroke recovery. J Neurosci.

[24] Milner TE (1992). A model for the generation of movements requiring endpoint precision. Neuroscience.

[25] Chen HM, Chen CC, Hsueh IP, Huang SL, Hsieh CL (2009). Test-retest reproducibility and smallest real difference of 5 hand function tests in patients with stroke. Neurorehabil Neural Repair.

[26] Folstein MF, Folstein SE, McHugh PR (1975). “Mini-mental state”. A practical method for grading the cognitive state of patients for the clinician. J Psychiatr Res.

[27] Lang JI (1983). Ein neuer Stereotest. Klin Mbl Augenheilk.

[28] Brown S, Weih L, Mukesh N, McCarty C, Taylor H (2001). Assessment of adult stereopsis using the Lang 1 Stereotest: a pilot study. Binocul Vis Strabismus Q.

[29] Schädler S, Kool J, Lüthi H, Marks D, Oesch P, Pfeffer A (2011). Assessments in der Rehabilitation.

[30] Mathiowetz V, Weber K, Volland G, Kashman N (1984). Reliability and validity of grip and pinch strength evaluations. J Hand Surg Am.

[31] Norman GR, Streiner DL (2008). Biostatistics: The bare essentials.

[32] Munro BH (2005). Statistical methods for health care research.

[33] Landis JR, Koch GG (1977). The measurement of observer agreement for categorical data. Biometrics.

[34] Weir JP (2005). Quantifying test-retest reliability using the intraclass correlation coefficient and the SEM. J Strength Cond Res.

[35] Shrout PE, Fleiss JL (1979). Intraclass correlations: Uses in assessing rater reliability. Psychological Bulletin.

[36] de Vet HC, Terwee CB, Knol DL, Bouter LM (2006). When to use agreement versus reliability measures. J Clin Epidemiol.

[37] Arnall FA, Koumantakis GA, Oldham JA, Cooper RG (2002). Between-days reliability of electromyographic measures of paraspinal muscle fatigue at 40, 50 and 60 % levels of maximal voluntary contractile force. Clin Rehabil.

[38] Denegar CR, Ball DW (1993). Assessing reliability and precision of measurement: an introduction to lntraclass correlation and standard error of measurement. J Sport Rehabil.

[39] Rankin G, Stokes M (1998). Reliability of assessment tools in rehabilitation: an illustration of appropriate statistical analyses. Clin Rehabil.

[40] Bland JM, Altman DG (1986). Statistical methods for assessing agreement between two methods of clinical measurement. Lancet.

[41] Bland JM, Altman DG (2003). Applying the right statistics: analyses of measurement studies. Ultrasound Obstet Gynecol.

[42] Liaw LJ, Hsieh CL, Lo SK, Chen HM, Lee S, Lin JH (2008). The relative and absolute reliability of two balance performance measures in chronic stroke patients. Disabil Rehabil.

[43] Brehm MA, Scholtes VA, Dallmeijer AJ, Twisk JW, Harlaar J (2012). The importance of addressing heteroscedasticity in the reliability analysis of ratio-scaled variables: an example based on walking energy-cost measurements. Dev Med Child Neurol.

[44] Bland JM, Altman DG (1996). Transforming data. BMJ.

[45] Euser AM, Dekker FW, le Cessie S (2008). A practical approach to Bland-Altman plots and variation coefficients for log transformed variables. J Clin Epidemiol.

[46] Aaronson N, Alonso J, Burnam A, Lohr KN, Patrick DL, Perrin E (2002). Assessing health status and quality-of-life instruments: attributes and review criteria. Qual Life Res.

[47] Bowler M, Amirabdollahian F, Dautenhahn K (2011). Using an embedded reality approach to improve test reliability for NHPT tasks. IEEE Int Conf Rehabil Robot.

[48] Terwee CB, Bot SD, de Boer MR, van der Windt DA, Knol DL, Dekker J (2007). Quality criteria were proposed for measurement properties of health status questionnaires. J Clin Epidemiol.

[49] Zou GY (2012). Sample size formulas for estimating intraclass correlation coefficients with precision and assurance. Stat Med.

[50] Jette AM, Tao W, Norweg A, Haley S (2007). Interpreting rehabilitation outcome measurements. J Rehabil Med.

[51] Schmidheiny A, Swanenburg J, Straumann D, de Bruin ED, Knols RH (2015). Discriminant validity and test re-test reproducibility of a gait assessment in patients with vestibular dysfunction. BMC Ear Nose Throat Disord.

[52] Information Brochure Armon Elemento. [http://www.armonproducts.com/images/Information_Brochure_Armon_Elemento.pdf].

[53] Altman DG. Practical statistics for medical research, [First CRC Press repr.] edn: Boca Raton: Chapman & Hall/CRC; 1999.

[54] Pollock A, Farmer SE, Brady MC, Langhorne P, Mead GE, Mehrholz J (2014). Interventions for improving upper limb function after stroke. Cochrane Database Syst Rev.

